# Supplementary material for: ASPSCR1::TFE3 orchestrates the angiogenic program of alveolar soft part sarcoma
Source: Nat Commun. 2023 Apr 7;14:1957. doi: 10.1038/s41467-023-37049-z (PMC10082046; doi:10.1038/s41467-023-37049-z)
Supplement: Supplementary file 1 — Supplementary Information [file 41467_2023_37049_MOESM1_ESM.pdf]

Supplementary Information

ASPSCR1-TFE3 orchestrates the angiogenic program of alveolar soft part sarcoma

Miwa Tanaka, Surachada Chuaychob, Mizuki Homme, Yukari Yamazaki, Ruyin Lyu, Kyoko Yamashita, Keisuke Ae, Seiichi Matsumoto, Kohei Kumegawa, Reo Maruyama, Wei Qu, Yohei Miyagi, Ryuji Yokokawa, Takuro Nakamura

6 Supplementary Figures with legends

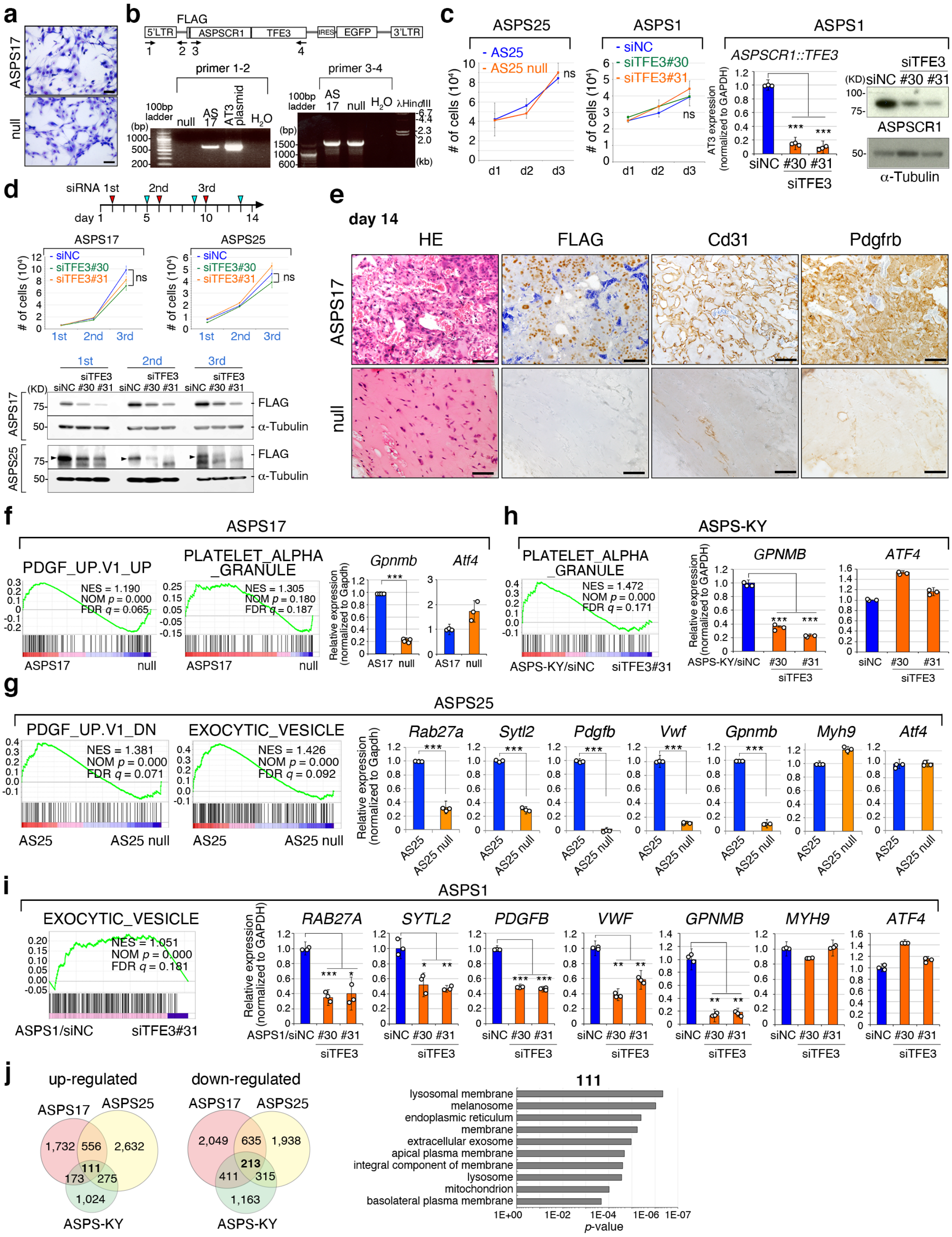

Supplementary Fig. 1 Reduced *ASPSCR1-TFE3* expression alters gene expression profiles in murine and human ASPS cells. a Photomicrographs of ASPS17 and null cells in culture. Scale bar: 50 μm. **b** Deletion of the DNA sequence including 5'-long terminal repeat (LTR) within the retroviral vector in ASPS null cells. The structure of pMY-ASPSCR1-TFE3-IRES-GFP (top), and genomic PCR

results for the 5'-LTR (bottom left) and the ASPSCR1-TFE3 sequence (bottom right) are shown. Experiments represent three biological replicates. **c** Cell proliferation of mouse ASPS25 and ASPS25 null cells (left), and human ASPS1 cells with siRNA treatment (center) (*n* = 3 per group). ns: no significance. The efficiency of *ASPSCR1::TFE3* knockdown is shown at transcriptional (*n* = 3 per group) and protein levels (right, representative immunoblots from three independent experiments). **d** *ASPSCR1::TFE3* knockdown in ASPS17 and ASPS25 cells. siRNAs for *TFE3* were given on days 2, 6, and 10 (red triangles), and the cell number was measured on days 5, 9, and 13 (blue triangles) (top) TFE3 knockdown did not affect significant effects on cell proliferation (middle) (*n* = 3 per group). ns: no significance. The efficiency of *ASPSCR1::TFE3* knockdown is shown at transcriptional and protein levels (bottom, representative immunoblots from two independent experiments). **e** Histology of transplanted area with ASPS17 and ASPS null cells 14 days after transplantation. Hematoxylin and eosin (HE) staining and immunohistochemistry with indicated antibodies. Significant reduction of FLAG-positive tumor cells, Cd31-positive endothelial cells, and Pdgfrb-positive pericytes in recipients with ASPS null cells. Scale bar: 50 μm. Experiments represent three biological replicates. **f** GSEA showing enrichment of PDGF and platelet alpha granule pathways between ASPS17 and null cells (left). qRT-PCR shows downregulation of *Gpnmb* in ASPS null cells while *Atf4* expression was increased (right) (*n* = 3 per group). **g** GSEA showing enrichment of PDGF and exocytic vesicle pathways between AS25 and AS25 null cells (left). Quantitative RT-PCR (qRT-PCR) showing downregulation of *Rab27a, Sytl2, Pdgfb*, *Vwf,* and *Gpnmb* in AS25 null cells while *Myh9* and expression was increased (right) (*n* = 3 per group). **h** GSEA showing enrichment of the platelet alpha granule pathways by comparing human ASPS-KY cells with and without knockdown of ASPSCR1::TFE3 (left). *GPNMB* is downregulated by the knockdown of *ASPSCR1::TFE3* while *ATF4* expression was unchanged (right) (*n* = 3 per group). **i** GSEA showing enrichment of the exocytic vesicle pathway by comparing human ASPS1 cells with and without knockdown of ASPSCR1::TFE3 (left). Downregulation of *RAB27A, SYTL2, PDGFB*, *VWF,* and *GPNMB* is shown (*n* = 3 per group). **j** Venn diagrams showing up-regulated and down-regulated genes in ASPS17, ASPS25 and human ASPS-KY cells upon *ASPSCR1::TFE3* expression (left). Enrichment of Gene Ontology analysis cellular process for 111 common up-regulated genes, showing inclusion of melanosome, extracellular exosome, and lysosome pathways (right). Statistical analyses in (**c, d, f, g, h, i**) were performed by two-sided Student's *t* test and *marks adjusted *P* value <0.05, **marks adjusted *P* value <0.01, ***marks adjusted *P* value <0.001 and ns marks no significance. The data presented as mean ± SD.

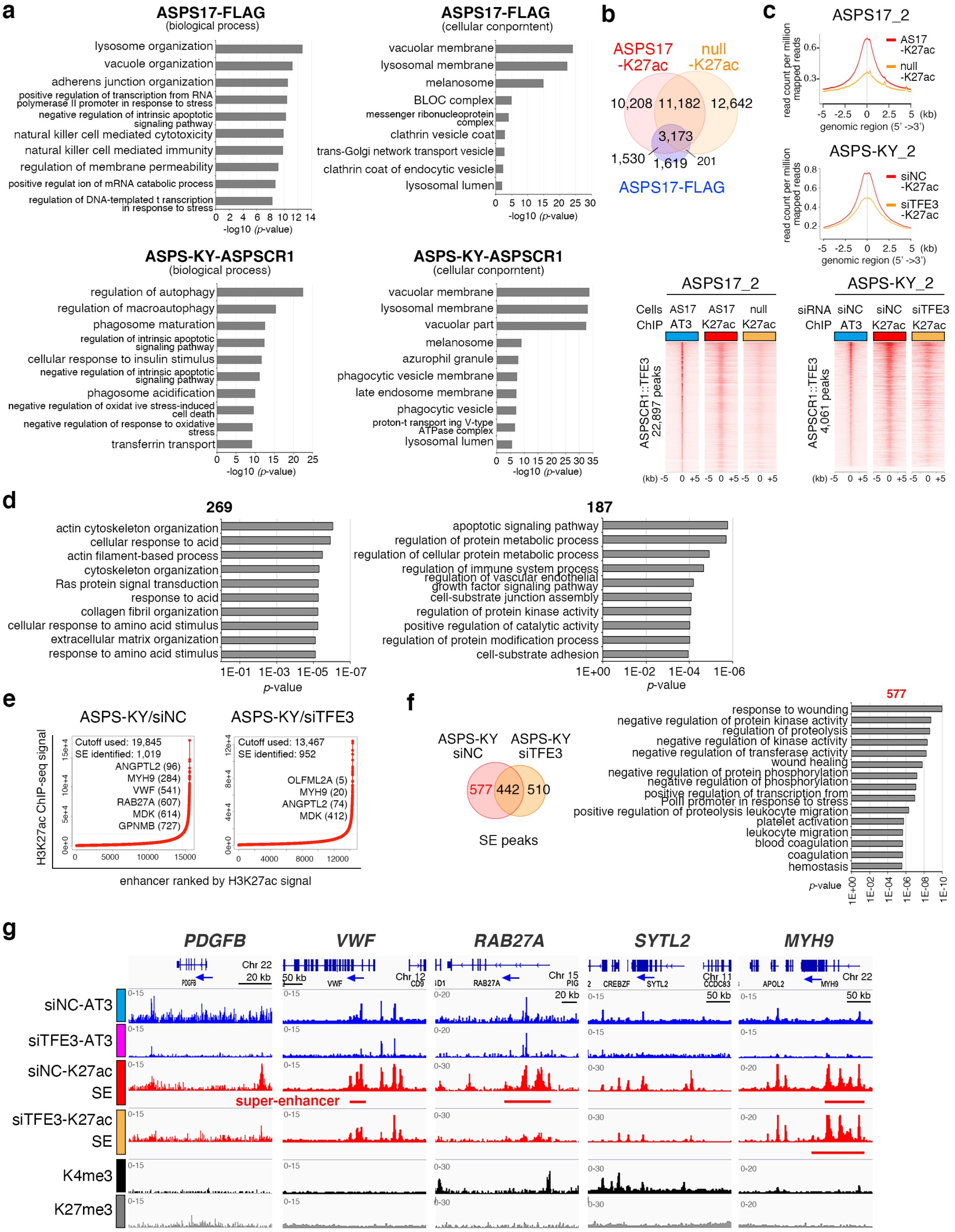

Supplementary Fig. 2 The loss of *ASPSCR1-TFE3* expression modulates distribution of super-enhancers. a The GREAT pathway analysis for ASPSCR1-TFE3 binding peaks in ASPS17 cells (top two graphs) and ASPS-KY cells (bottom two graphs). *P* values are calculated using a binominal test. **b** Venn diagram showing the number and overlapping of H3K27ac in ASPS17 and null cells, and ASPSCR1-TFE3 (FLAG) in ASPS17 cells. **c** Composite plots of replicates to Fig.

2d showing a significant reduction in H3K27ac signals in the absence of ASPSCR1::TFE3 in both mouse and human ASPS (top). Heat maps showing ASPSCR1::TFE3 and H3K27ac signals in murine ASPS17 and human ASPS-KY cells. Reduction in H3K27ac signals is observed in both cells (bottom). **d** The GREAT pathway analysis for 269 overlapping in both ASPS17 and null cells, and 187 null-specific super-enhancers appeared in Fig. 2f. *P* values are calculated using a binominal test. **e** Enhancers are ranked by increasing H3K27ac signals in ASPS-KY cells introduced with control siRNA and siTFE3. Using the ROSE algorithm, 1019 and 952 enhancers were defined as SEs in ASPS-KY cells with control siRNA and siTFE3, respectively. **f** Venn diagram showing overlapping and distinct SEs (left). Enrichment of Gene Ontology biological process for 577 *ASPSCR1-TFE3*-positive ASPS-KY specific SEs (right). *P* values are calculated using a binominal test. **g** ChIP-seq track at *PDGFB, VWF, RAB27A, SYTL2,* and *MYH9* genomic loci, showing association between ASPSCR1::TFE3 and H3K27ac binding in ASPS-KY cells. Significant loss of H3K27ac signals and/or SE by *TFE3* knockdown are exhibited. The *MYH9* genomic locus is shown as an example of un-affected SEs by *ASPSCR1::TFE3* knockdown.

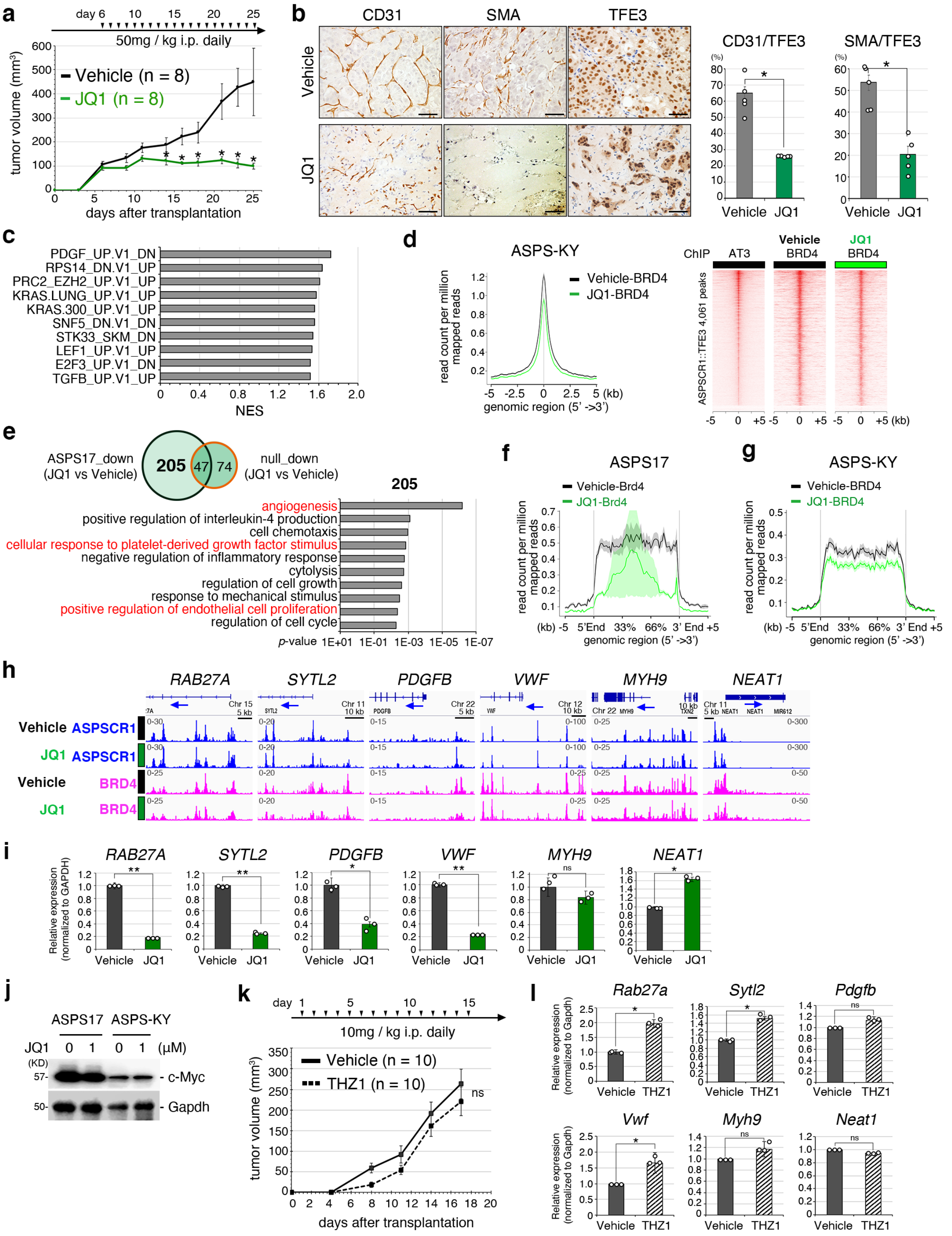

Supplementary Fig. 3 JQ1 suppresses angiogenesis-associated gene expression via suppression of Brd4 binding whereas THZ1 does not affect angiogenesis and tumor development of ASPS. a Suppression of *in vivo* tumor growth of human ASPS-KY by JQ1 treatment. Growth curves of the transplanted tumors and treated with JQ1 or vehicle and the experimental schedule are shown ($n = 4$ mice/8 independent tumors per group). The tumor volume

was measured using 2 tumors per mouse. **b** Immunohistological examination of the ASPS-KY tumor samples treated with JQ1 or vehicle. Anti-CD31 (endothel), anti-α-SMA (pericyte) and TFE3 (tumor cell) were used. CD31- and α-SMA-positive areas were measured and quantitated using the image J software. Positive areas were normalized by the number of TFE3-positive cells (right) (*n* = 5 independent areas). scale bar: 50 μm. Experiments represent three biological replicates. **c** GSEA results on the comparison between ASPS17 cells treated with vehicle and JQ1 gene expression signatures. Top 10 pathways for normalized enrichment scores are indicated. **d** Composite plots showing a significant reduction in Brd4-binding signals around ASPSCR1::TFE3-binding peaks by JQ1 treatment (0.5 μM for 48 hr) on ASPS-KY cells (left). Heat maps showing ASPSCR1::TFE3 and BRD4 with or without JQ1 treatment, in human ASPS-KY cells (right). **e** Venn diagram showing overlapping and distinct down-regulated genes in ASPS17 and null cells treated with JQ1 and vehicle (top). The DAVID analysis for 205 downregulated genes unique in ASPS17 cells showing enrichment of angiogenesis-associated pathways (bottom, indicated in red). **f** Composite plots showing the significant reduction of Brd4 binding signals around 527 SEs of ASPS17 cells by JQ1 treatment. The data presented as mean ± SD. **g** Composite plots showing the significant reduction of Brd4 binding signals around 1019 SEs of ASPS-KY cells by JQ1 treatment. The data presented as mean ± SD. **h** ChIP-seq track at *RAB27A, SYTL2, PDGFB, VWF, MYH9,* and *NEAT1* genomic loci with ASPSCR1::TFE3-binding peaks in ASPS-KY cells. Mild reduction of BRD4 signals at *RAB27A, SYTL2, PDGFB,* and *VWF* loci, with BRD4 signlas at *MYH9* and *NEAT1* loci remaining unchanged. ASPSCR1::TFE3 signals remained unchanged by JQ1 treatment. **i** qRT-PCR showing downregulation of *RAB27A, SYTL2, PDGFB* and *VWF* in ASPS-KY treated with JQ1, with the expression of *MYH9* and *NEAT1* was not downregulated (*n* = 3 per group). **j** Western blotting showing expression of c-Myc. c-Myc expression was not reduced by JQ1 treatment in ASPS17 and ASPS-KY cells. Data are presented by two independent experiments. **k** Effects of THZ1 treatment on the *in vivo* growth of ASPS17 cells (*n* = 5 mice/10 independent tumors per group). The tumor volume was measured using 2 tumors per mouse. **l** Expression of *Rab27a, Sytl2, Pdgfb, Vwf, Myh9, Neat1,* and *Atf4* in ASPS17 treated with 0.5 μM of THZ1 for 48 hr. None of genes show downregulated expression (*n* = 3 per group). Statistical analyses in (**a, b, i, k, l**) were performed by two-sided Student's *t* test and *marks adjusted *P* value <0.05, **marks adjusted *P* value <0.01, ***marks adjusted *P* value <0.001 and ns marks no significance. The data presented as mean ± SD. Source data are provided as a Source Data file.

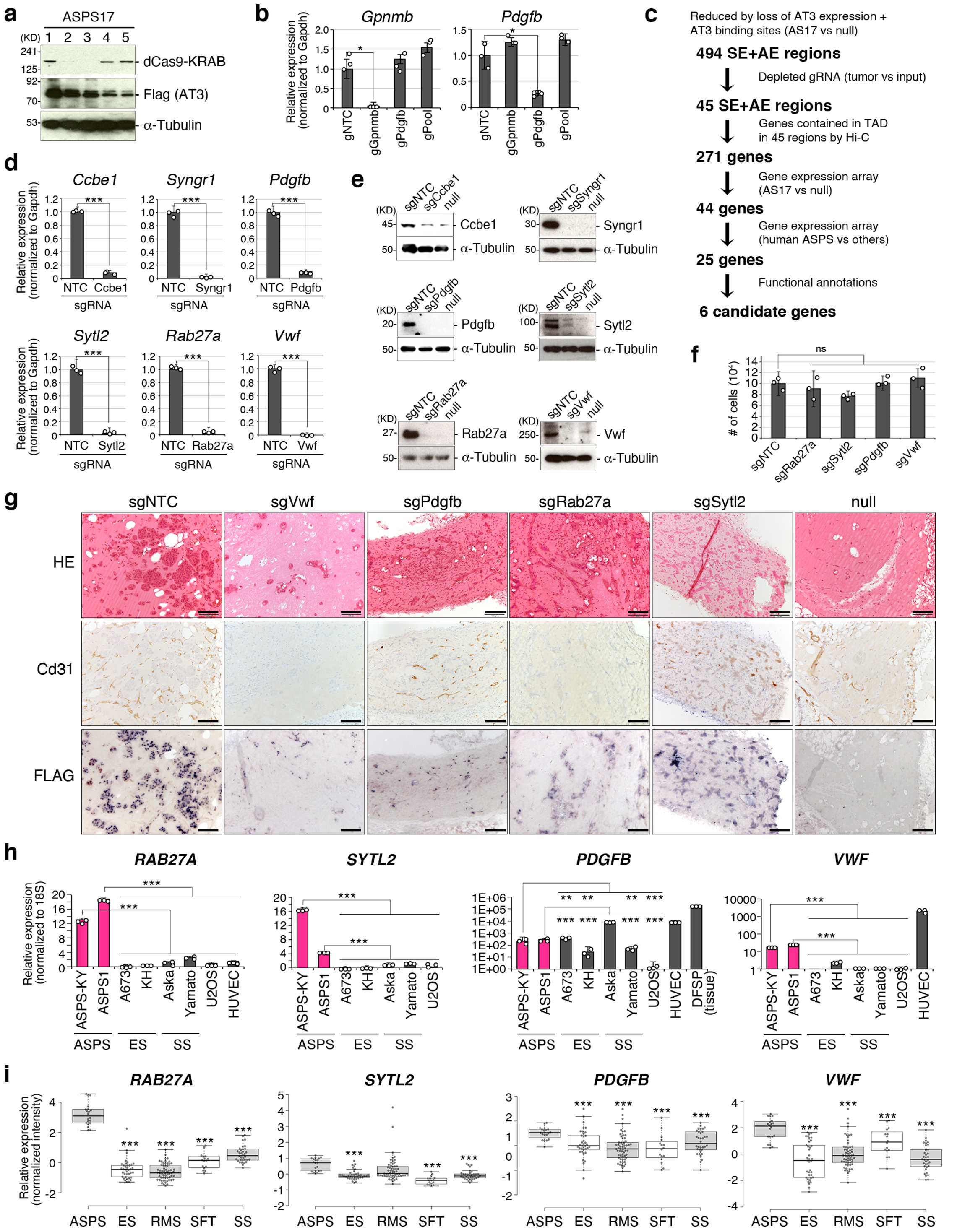

Supplementary Fig. 4 Identification of ASPSCR1::TFE3 targets by epigenomic CRISPR/dCas9 screening. a Exogenous expression of dCas9-KRAB protein in ASPS17 cells detected with the anti-Cas9 antibody. The clone #1 was used for the subsequent screening. Data are presented by two independent experiments. **b** Efficiencies of downregulated expression by introducing individual gRNA for *Gpnmb* and *Pdgfb* in dCas9-KRAB-expressing ASPS17 cells (*n*

= 3 per group). * $P < 0.05$, ** $P < 0.01$. **c** The schematic diagram of the screening procedure. A total of 494 SE and active enhancers (AE) were selected as primary targets. **d** Validation of gene knockout confirmed by qRT-PCR ($n = 3$ per group). *** $P < 0.001$. **e** Validation of gene knockout confirmed by western blotting. Data are presented by three independent experiments. **f** Knockout of *Rab27a, Sytl2, Pdgfb,* or *Vwf* does not suppress cell proliferation *in vitro* ($n = 3$ per group). **g** Immunostaining for Cd31 and FLAG (ASPSCR1::TFE3) in recipient nude mice two weeks after transplantation of ASPS cells showing significant suppression of angiogenesis and tumor growth by knockout of *Vwf, Pdgfb, Rab27a* and *Sytl2* as well as in the *ASPSCR1-TFE3* loss (null cells). Scale bar, 100 µm. Experiments represent three biological replicates. **h** qRT-PCR of *RAB27A, SYTL2, PDGFB*, and *VWF* in human sarcoma cell lines ($n = 3$ per group). * $P < 0.05$, *** $P < 0.001$. **i** Upregulated expression of *RAB27A, SYTL2, PDGFB*, and *VWF* in ASPS among five human sarcoma types, with 20 cases of ASPS, 37 Ewing sarcoma (ES), 58 rhabdomyosarcoma (RMS), 16 solitary fibrous tumor (SFT), and 34 synovial sarcoma (SS). The mean of the normalized signal intensity values for each gene was calculated using GeneSpring GX 14.9. The boxes show the interquartile range, with the median marked as a horizontal band. Whiskers represent the highest (lowest) datapoint within 1.5 times the interquartile range of the 75th (25th) percentile. The dots represent each datapoint. Statistical analyses in (**b, f, h, i**) were performed by one-way ANNOVA and in (**d**) were performed by Student's *t* test and *marks adjusted *P* value <0.05, **marks adjusted *P* value <0.01, ***marks adjusted *P* value <0.001 and ns marks no significance. The data presented as mean ± SD. Box plots presented as median ± interquartile range. Source data are provided as a Source Data file.

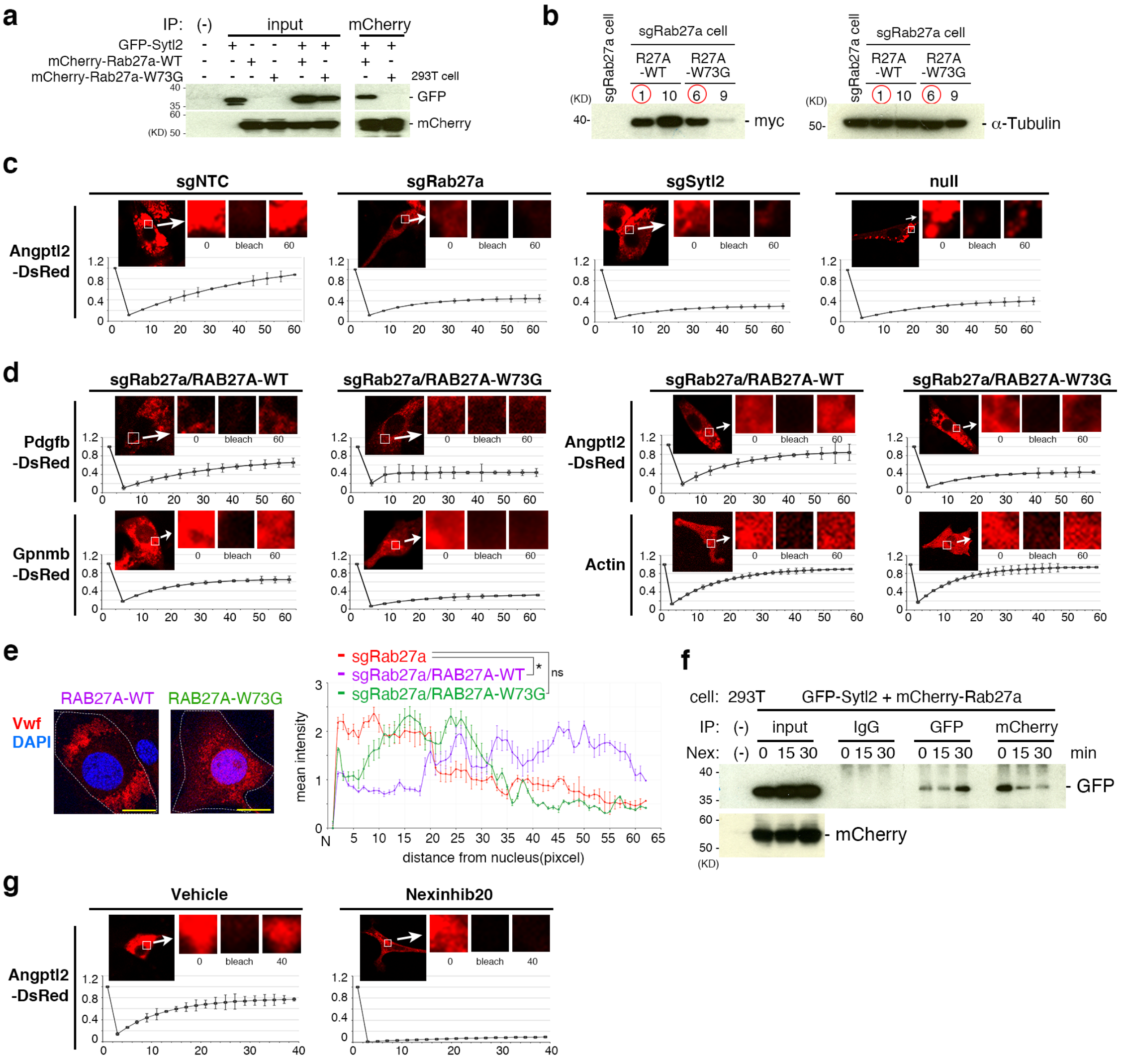

Supplementary Fig. 5 Intracellular trafficking of angiogenic factors is promoted by the Rab27a/Sytl2 axis. a Co-immunoprecipitation showing interaction between Sytl2 and wild type Rab27a but not the W73G mutant of Rab27a. GFP-tagged Sytl2 and mCherry-tagged Rab27a were introduced into HEK293T cells. Data are presented by three independent experiments. **b** Western blotting showing exogenous expression of myc-tagged wild type and W73G RAB27A in ASPS17 cells with knockout of endogenous Rab27a. Red circles indicate clones used for ELISA and the trafficking experiment shown in Fig. 5a, e and Supplementary Fig. 5d, e. Data are presented by three independent experiments. **c** FRAP showing marked delay in trafficking of DsRed-labeld Angptl2 by *Rab27a* knockout, *Sytl2* knockout and ASPS null cells. Representative images of FRAP of ASPS17 cells are shown in upper panels and representative normalized traces of FRAP for each experiment are shown in lower panels. Each point represents one image acquired every 5 min (n = 3 per group). The data presented as mean ± SD. **d** FRAP showing

rescue of trafficking by wild type RAB27A but not W73G mutant in ASPS17 cells with endogenous *Rab27a* knockout. Time course of DsRed-labeld Pdgfb, Gpnmb, and Angptl2 distributions are shown (*n* = 3 per group). The data presented as mean ± SD. **e** Immunofluorescence showing intracellular localization of Vwf in Rab27a knockout cells in the presence of exogenous wild type or W73G proteins. Localization in peripheral areas in ASPS17 cells with *Rab27a* knockout was recovered by introduction of human wild type RAB27A but not by the W73G mutant (left). Intracellular distribution of Vwf in each cell type was plotted as mean intensities of fluorescence at indicated distances from nucleus (right) (*n* = 3 per group). Statistical analyses were performed by one-way ANOVA and *marks adjusted *P* value <0.05. Scale bar, 10 μm. Experiments represent three biological replicates. **f** Inhibition of Sytl2 and Rab27a interaction by Nexinhib 20 shown by co-immunoprecipitation. HEK293T cells were introduced with mCherry-tagged Rab27a and GFP-tagged Sytl2. Cells were treated with 10 μM of Nexinhib 20 and were subjected to immunoprecipitation at indicated time points. Significant immunoprecipitated signals for GFP-Sytl2 are shown at 15 and 30 min after treatment. Data are presented by three independent experiments. **g** FRAP showing significant delay in Angptl2 trafficking by Nexinhib 20 treatment (*n* = 3 per group). The data presented as mean ± SD.

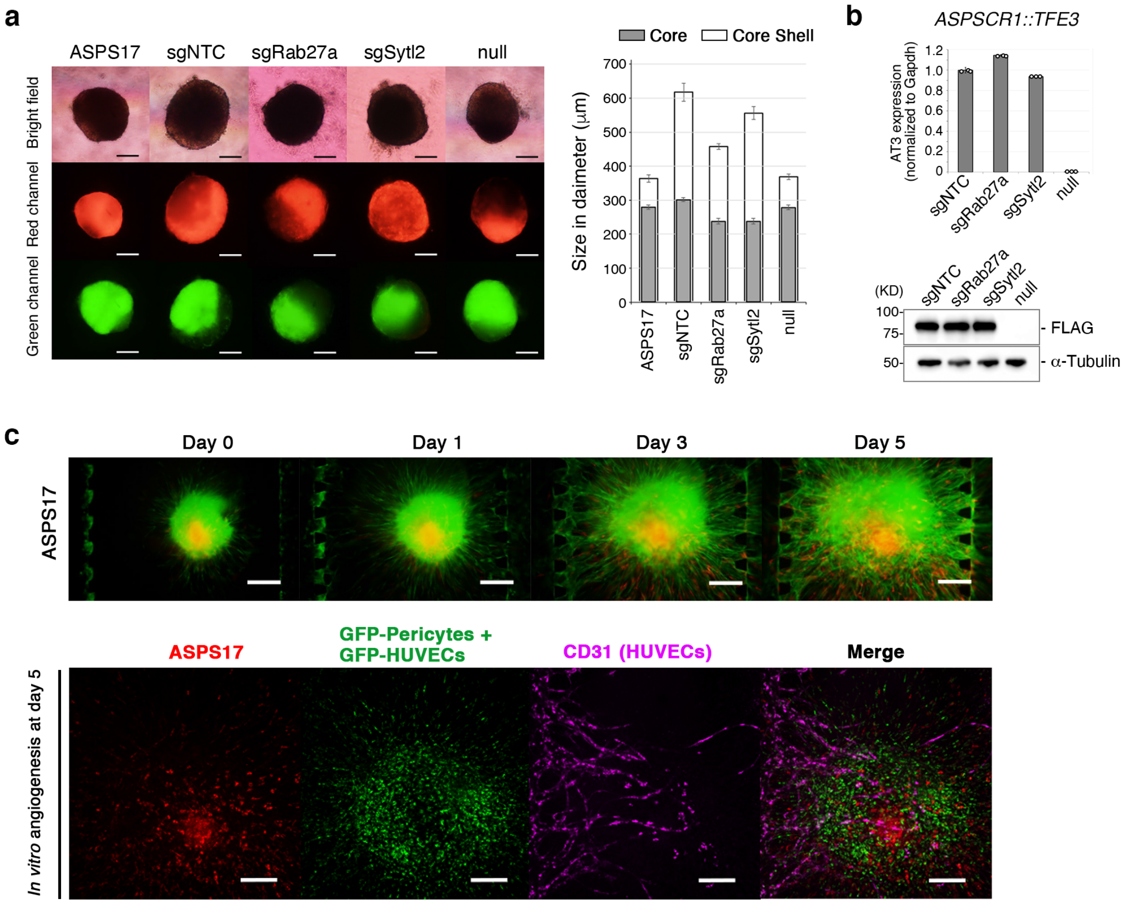

Supplementary Fig. 6 Induction of vascular network by co-culture of tumor spheroids, pericytes and HUVEC cells in the microfluidic device. a Core shell spheroid structure. The bright field, green fluorescence channel, and red fluorescence channel images of the co-culture core shell spheroids after three days in suspension culture (left). The size in diameter of core and core shell spheroids was measured ($n = 20$ per group) (right). The data presented as mean ± SD. Scale bar, 200 μm. Experiments represent three replicates. **b** Expression of ASPSCR1::TFE3 in each spheroid was confirmed at transcription (top, n = 3 per group) and protein level (bottom, representative immunoblots from three independent experiments). The data presented as mean ± SD. **c** The time-course fluorescent images of the *in vitro* angiogenesis using the core shell ASPS17 cells and the immunofluorescence-based characterization on day 5. Scale bar, 200 μm. Experiments represent three biological replicates.
