# Supplementary material for: ASPSCR1::TFE3 orchestrates the angiogenic program of alveolar soft part sarcoma
Source: Nat Commun. 2023 Apr 7;14:1957. doi: 10.1038/s41467-023-37049-z (PMC10082046; doi:10.1038/s41467-023-37049-z)
Supplement: Supplementary file 3 — Description of Additional Supplementary Files [file 41467_2023_37049_MOESM3_ESM.pdf]

Description of Additional Supplementary Files

File Name: Supplementary Data 1
Description: Genes upregulated in ASPS17 cells compared with ASPS null cells.

File Name: Supplementary Data 2
Description: Genes downregulated in ASPS17 cells compared with ASPS null cells.

File Name: Supplementary Data 3
Description: Genes downregulated in ASPS-KY cells by ASPSCR1-TFE3 knockdown.

File Name: Supplementary Data 4
Description: Genes upregulated in ASPS-KY cells by ASPSCR1-TFE3 knockdown.

File Name: Supplementary Data 5
Description: Super-enhancers (SEs) specific for ASPS17 cells.

File Name: Supplementary Data 6
Description: Common SEs in ASPS17 and ASPS null cells.

File Name: Supplementary Data 7
Description: SEs specific for ASPS null cells.

File Name: Supplementary Data 8
Description: Target enhancers and the list of 7716 gRNAs used for epigenomic CRISPR screening.

File Name: Supplementary Data 9
Description: The MAGeCK result of CRISPR screening.

File Name: Supplementary Data 10
Description: Genes included 45 enhancer regions.

File Name: Supplementary Data 11
Description: Differential expression of 271 genes in mouse and human ASPS.

File Name: Supplementary Data 12
Description: Oligonucleotide sequences for PCR primers.

File Name: Supplementary Data 13
Description: A list of oligonucleotide sequences used for CRISPR screening and gene editing.
