# Supplementary material for: ASPSCR1::TFE3 orchestrates the angiogenic program of alveolar soft part sarcoma
Source: Nat Commun. 2023 Apr 7;14:1957. doi: 10.1038/s41467-023-37049-z (PMC10082046; doi:10.1038/s41467-023-37049-z)
Supplement: Supplementary file 17 — Reporting Summary [file 41467_2023_37049_MOESM17_ESM.pdf]

nature portfolio

Corresponding author(s): Takuro Nakamura

Last updated by author(s): Jan 18, 2023

Reporting Summary

Nature Portfolio wishes to improve the reproducibility of the work that we publish. This form provides structure for consistency and transparency in reporting. For further information on Nature Portfolio policies, see our Editorial Policies and the Editorial Policy Checklist.

Statistics

For all statistical analyses, confirm that the following items are present in the figure legend, table legend, main text, or Methods section.

n/a	Confirmed	
☐	☒	The exact sample size (*n*) for each experimental group/condition, given as a discrete number and unit of measurement
☐	☒	A statement on whether measurements were taken from distinct samples or whether the same sample was measured repeatedly
☐	☒	The statistical test(s) used AND whether they are one- or two-sided<br>*Only common tests should be described solely by name; describe more complex techniques in the Methods section.*
☒	☐	A description of all covariates tested
☒	☐	A description of any assumptions or corrections, such as tests of normality and adjustment for multiple comparisons
☐	☒	A full description of the statistical parameters including central tendency (e.g. means) or other basic estimates (e.g. regression coefficient) AND variation (e.g. standard deviation) or associated estimates of uncertainty (e.g. confidence intervals)
☐	☒	For null hypothesis testing, the test statistic (e.g. *F*, *t*, *r*) with confidence intervals, effect sizes, degrees of freedom and *P* value noted<br>*Give P values as exact values whenever suitable.*
☒	☐	For Bayesian analysis, information on the choice of priors and Markov chain Monte Carlo settings
☒	☐	For hierarchical and complex designs, identification of the appropriate level for tests and full reporting of outcomes
☒	☐	Estimates of effect sizes (e.g. Cohen's *d*, Pearson's *r*), indicating how they were calculated

Our web collection on statistics for biologists contains articles on many of the points above.

Software and code

Policy information about availability of computer code

Data collection	ZEN 2009 (Zeiss) - image acquisition, MAS 5.0 (Affymetrix) - microarray data acquisition, Samtools 1.2 - ChiP-seq alignment, Juicer - Hi-C alignment
Data analysis	Image J ver 2.0; GeneSpring GX ver 14.9; GSEA-P 2.0; IPA 2021 or earlier; MACS ver 1.4; Cistrome (http://cistrome.org/ap/root); Nucleus (https://rias.rhelixa.com);<br>NGSPlot ver 2.47.1 (https://anaconda.org/bioconda/r-ngsplot); IGV ver 2.3.80; HOMER ver 4.11.1; Rank Ordering of Super-Enhancers (ROSE) (http://younglab.wi.mit.edu/super_enhancer_code.html); GREAT ver 4.0.4;<br>DESeq2 ver3.12 (https://bioconductor.org/packages/release/bioc/html/DESeq2.html); Juicebox ver 2.2.6 (https://www.aidenlab.org/juicebox); Skewer ver 0.2.2; featureCounts ver 2.0.10; glmOLFTest ver 3.32.1;<br>MAGeCK ver 0.5.9 (https://sourceforge.net/p/mageck/wiki/Home/); MAGeCKFlute ver 3.16 (https://bioconductor.org/packages/release/bioc/html/MAGeCKFlute.html); STAR ver 2.7.8a (https://code.google.com/archive/p/rna-star)

For manuscripts utilizing custom algorithms or software that are central to the research but not yet described in published literature, software must be made available to editors and reviewers. We strongly encourage code deposition in a community repository (e.g. GitHub). See the Nature Portfolio guidelines for submitting code & software for further information.

Data

Policy information about availability of data

All manuscripts must include a data availability statement. This statement should provide the following information, where applicable:

- Accession codes, unique identifiers, or web links for publicly available datasets
- A description of any restrictions on data availability
- For clinical datasets or third party data, please ensure that the statement adheres to our policy

The raw RNA-seq and ChIP-seq data generated in this study have been deposited in the NCBI Gene Expression Omnibus (GEO) database (http://www.ncbi.nlm.nih.gov/geo) under the accession number GSE215265 (https://www.ncbi.nlm.nih.gov/geo/query/acc.cgi?acc=GSE215265) and GSE189163 (https://www.ncbi.nlm.nih.gov/geo/query/acc.cgi?acc=GSE189163), respectively. The raw Hi-C data generated in this study have been deposited in the DDBJ Sequenced Read Archive under the accession numbers DRR330423 (https://ddbj.nig.ac.jp/resource/sra-run/DRR330423) and DRR330424 (https://ddbj.nig.ac.jp/resource/sra-run/DRR330424). The reused publicly available data for gene expression are available in the NCBI Gene Expression Omnibus (GEO) database (http://www.ncbi.nlm.nih.gov/geo) under the accession number GSE12102 (https://www.ncbi.nlm.nih.gov/geo/query/acc.cgi?acc=GSE12102), GSE13433 (https://www.ncbi.nlm.nih.gov/geo/query/acc.cgi?acc=GSE13433), GSE20196 (https://www.ncbi.nlm.nih.gov/geo/query/acc.cgi?acc=GSE20196), GSE32569 (https://www.ncbi.nlm.nih.gov/geo/query/acc.cgi?acc=GSE32569), GSE66533 (https://www.ncbi.nlm.nih.gov/geo/query/acc.cgi?acc=GSE66533), and in the EMBL-EBI ArrayExpress (https://www.ebi.ac.uk/biostudies/arrayexpress) under the accession number E-MTAB-1361 (https://www.ebi.ac.uk/biostudies/arrayexpress/studies/E-MTAB-1361?query=E-MTAB-1361). The following human genome accessions were used: The NCBI accession of the UCSC hg19 genome is GCA_000001405.1. The following mouse genome accessions were used: GRCM37/mm9 assembly via UCSC under GCA_000001635.1. The remaining data are available within the Article, Supplementary Information or Source Data file.

Human research participants

Policy information about studies involving human research participants and Sex and Gender in Research.

Reporting on sex and gender	Patients were consecutively included in this study, therefore there were no selection of patients based on sex in this study. 20 females and 17 males were included in this study. Gender of participants was not intentionally selected, because we did not find association of the sex and gene expression profiles in sarcoma patient samples.
Population characteristics	All patient samples (age ranging five to 87 year old) were obtained by Japanese Foundation for Cancer Research in accordance with the Declaration of Helsinki after written consent from the patients upon enrolling in the trial.
Recruitment	Patients did not receive compensation for participation in this study.
Ethics oversight	Informed consent was obtained from donors, and the study was approved by Institutional Review Board at the Japanese Foundation for Cancer Research under license 2013-1155.

Note that full information on the approval of the study protocol must also be provided in the manuscript.

Field-specific reporting

Please select the one below that is the best fit for your research. If you are not sure, read the appropriate sections before making your selection.

☒ Life sciences ☐ Behavioural & social sciences ☐ Ecological, evolutionary & environmental sciences

For a reference copy of the document with all sections, see nature.com/documents/nr-reporting-summary-flat.pdf

Life sciences study design

All studies must disclose on these points even when the disclosure is negative.

Sample size	No statistical analyses were performed to pre-determine the sample size. Microarray and ChIP-seq experiments were performed with three and two technical replicates, respectively, and these analyses were sufficiently to identify enhancer modifications in ASPS samples. For all other experiments, the sample size was used in accordance with standard rigor and reproducibility practice guidlines by NIH and is indicated in the figure legends. The sample size is indicated in the figure legends.
Data exclusions	No data were excluded from the study.
Replication	The majority of experiments other than ChIP-seq were repeated at least three independent times with similar results. ChIP-seq experiments were performed at least twice and reproducibility was confirmed. Where experiments were performed once, the phenotypes were validated using same experiment with different cell lines. The exact n for each experiment is described in the Figure Legends. Mouse studies were not replicated but included sufficient sample size to account for biological variability.
Randomization	Randomization was not applicable, because there was no comparison between different repeated experiments.

Blinding | Investigators are not blinded for the analysis of experiments since it was not feasible.

Reporting for specific materials, systems and methods

We require information from authors about some types of materials, experimental systems and methods used in many studies. Here, indicate whether each material, system or method listed is relevant to your study. If you are not sure if a list item applies to your research, read the appropriate section before selecting a response.

Materials & experimental systems

n/a	Involved in the study
☐	☒ Antibodies
☐	☒ Eukaryotic cell lines
☒	☐ Palaeontology and archaeology
☐	☒ Animals and other organisms
☒	☐ Clinical data
☒	☐ Dual use research of concern

Methods

n/a	Involved in the study
☐	☒ ChIP-seq
☒	☐ Flow cytometry
☒	☐ MRI-based neuroimaging

Antibodies

Antibodies used

ASPSCR1 (Sigma-Aldrich, HPA026749, 1:1000), mouse CD31 (Cell Signaling, 77699, 1:100), human CD31 (Abcam, ab28346, 1:100), PDGFRB (R&D Systems, BAF1042, 1:100), Histone H3K27ac (Active Motif, 39133, 1:100), Histone H3K4me3 (Abcam, ab8580, 1:100), Histone H3K27me3 (Millipore, 07-449, 1:100), FLAG (Sigma-Aldrich, F7425, 1:100 for ChIP and immunofluorescence), FLAG (Sigma-Aldrich, F3165, 1:1000 for Western blot and 1:100 for IHC), BRD4 (Bethyl Laboratories, A301-985A100, 1:100), Cas9 (Novus Biologicals, NBP2-36440, 1:1000), VWF (DAKO, N1505, 1:100 for immunofluorescence), RAB27A (Cell Signaling, 69295, 1:1000 for Western blot and 1:100 for IHC), SYTL2 (Santa Cruz Biotechnology, sc393847, 1:100 for IHC), SYTL2 (Proteintech, 12359, 1:1000 for Western blot), VWF (Santa Cruz Biotechnology, sc365712, 1:100 for IHC), VWF (Bioss, bs4754R, 1:1000 for Western blot), aSMA (DAKO, MO851, 1:100), NG2 (Millipore, AB5320, 1:100), TFE3 (Santa Cruz Biotechnology, sc5958, 1:100), c-Myc (Cell Signaling, 5605, 1:1000), CCBE1 (Affinity Biosciences, DF10092, 1:1000), SYNGR1 (Cell Signaling, 20874, 1:1000), PDGFB (Abcam, ab23914, 1:1000), Myc-tag (Santa Cruz Biotechnology, sc40, 1:1000), GFP (Merck Millipore, MAB3580, 1:1000), mCherry (Cell Signaling, 43590, 1:1000), DsRed (Clontech, 632496, 1:1000), a-Tubulin (Sigma-Aldrich, T5168, 1:1000), Gapdh (HyTest, 5G4, 1:1000), rabbit IgG conjugated with RRX (Jackson Immunoresearch, 111-295-144, 1:100), mouse IgG conjugated with HRP (Cytiva, NA931, 1:2000), and rabbit IgG conjugated with HRP (Cytiva, NA934, 1:2000).

Validation

The following antibodies were validated by the Takuro Nakamura lab using gene knockout and/or overexpression experiments: FLAG (Sigma-Aldrich, F3165, Cas9 (Novus Biologicals, NBP2-36440), RAB27A (Cell Signaling, 69295), SYTL2 (Proteintech, 12359), VWF (Bioss, bs4754R), CCBE1 (Affinity Biosciences, DF10092), SYNGR1 (Cell Signaling, 20874), PDGFB (Abcam, ab23914), Myc-tag (Santa Cruz Biotechnology, sc40), GFP (Merck Millipore, MAB3580), mCherry (Cell Signaling, 43590), and DsRed (Clontech, 632496).
The validation of following antibodies were shown in the following manufacturers' websites:
ASPSCR1: https://www.sigmaaldrich.com/JP/ja/product/sigma/hpa026749
mouse CD31: https://www.cellsignal.jp/products/primary-antibodies/cd31-pecam-1-d8v9e-xp-rabbit-mab/77699?_=1673936795263&Ntt=77699&tahead=true
human CD31: https://www.abcam.co.jp/phosphotyrosine-antibody-py20-rhodamine-ab28346.html
PDGFRB: https://www.rndsystems.com/products/mouse-pdgf-rbeta-biotinylated-antibody_baf1042
Histone H3K27ac: https://www.activemotif.jp/catalog/details/39133/histone-h3-acetyl-lys27-antibody-pab
Histone H3K4me3: https://www.abcam.co.jp/histone-h3-tri-methyl-k4-antibody-chip-grade-ab8580.html
Histone H3K27me3: https://www.sigmaaldrich.com/JP/ja/product/mm/07449
FLAG, F7425: https://www.sigmaaldrich.com/JP/ja/product/sigma/f7425
BRD4: https://www.fortislife.com/products/primary-antibodies/rabbit-anti-brd4-antibody/BETHYL-A301-985
SYTL2: https://datasheets.scbt.com/sc-393847.pdf
VWF: https://datasheets.scbt.com/sc-365712.pdf
aSMA: https://www.agilent.com/store/productDetail.jsp?catalogId=M085101-2
NG2: https://www.sigmaaldrich.com/JP/ja/product/mm/ab5320
c-Myc: https://www.cellsignal.jp/products/primary-antibodies/c-myc-d84c12-rabbit-mab/5605

Eukaryotic cell lines

Policy information about cell lines and Sex and Gender in Research

Cell line source(s)

Mouse ASPS and pericyte cell lines were derived in our lab as described in the Method section. ASPS-KY was established by Yohei Miyagi as described in ref. 53. ASPS1 was obtained from Robert Shoemaker (ref. 54). Aska and Yamato were obtained from Norifumi Naka (Naka et al., 2010). KH was established in our lab (Tanaka et al., 2014). A673, U2OS and HEK293T were purchased from ATCC (CRL-1598, HTB-96, and CRL-3216). HUVEC and human placental microvascular pericytes were purchased from Angio-proteomie (AP-0001GFP-PM and cAP-0029GFP).

Authentication

ASPS, Ewing sarcoma and synovial sarcoma cell lines were authenticated by the STR assay.

Mycoplasma contamination	All cell lines were tested negative for mycoplasma infection.
Commonly misidentified lines (See ICLAC register)	No commonly misidentified cell lines were used.

Animals and other research organisms

Policy information about studies involving animals; ARRIVE guidelines recommended for reporting animal research, and Sex and Gender in Research

Laboratory animals	For allograft experiments, 6-8 weeks old female Balb/c nude mice were used from Japan Clea. All animals were tested for pathogen free and in a healthy condition.
Wild animals	No wild animals were used in this study.
Reporting on sex	Only female mice were used. The studies of ours and others demonstrated that no sex predisposition in the incidence and prognosis of alveolar soft part sarcoma, validating our initial choice to use only one sex.
Field-collected samples	No field collected samples were used in this study.
Ethics oversight	All animal experiments described in this study were performed in strict accordance with standard ethical guidelines and were approved by the animal care committee at the Japanese Foundation for Cancer Research under licenses 10-05-9 and 0604-3-13.

Note that full information on the approval of the study protocol must also be provided in the manuscript.

ChIP-seq

Data deposition

- [x] Confirm that both raw and final processed data have been deposited in a public database such as GEO.
- [x] Confirm that you have deposited or provided access to graph files (e.g. BED files) for the called peaks.

Data access links *May remain private before publication.*	https://www.ncbi.nlm.nih.gov/geo/query/acc.cgi?acc=GSE189163
Files in database submission	GSM5695585 mASPS_ASPSCR1-TFE3_Flag, GSM5695586 mASPS_H3K4me3, GSM5695587 mASPS_H3K27ac, GSM5695588 mASPS_H3K27me3, GSM5695589 mASPS-Null_H3K27ac, GSM5695590 mASPS_JQ1_Brd4, GSM5695591 mASPS_DMSO_Brd4, GSM5695592 mASPS_Input, GSM5695593 hASPS_ASPSCR1-TFE3, GSM5695594 hASPS_H3K4me3, GSM5695595 hASPS_H3K27ac, GSM5695596 hASPS_H3K27me3, GSM5695597 hASPS_siTFE3_H3K27ac, GSM5695598 hASPS_siNC_H3K27ac, GSM5695599 hASPS_input, GSM6523675 mASPS_ASPSCR1-TFE3_Flag2, GSM6523676 mASPS_ASPSCR1-TFE3_Flag3, GSM6523677 mASPS null_ASPSCR1-TFE3_Flag, GSM6523678 mASPS_H3K27ac_2, GSM6523679 mASPS null_H3K27ac_2, GSM6523680 mASPS_ASPSCR1-TFE3_Flag_DMSO, GSM6523681 mASPS_ASPSCR1-TFE3_Flag_JQ1, GSM6523682 hASPS_ASPSCR1-TFE3_2, GSM6523683 hASPS_ASPSCR1-TFE3_3, GSM6523684 hASPS_siTFE3_ASPSCR1-TFE3, GSM6523685 hASPS_siTFE3_H3K27ac_2, GSM6523686 hASPS_siTFE3_H3K27ac_3, GSM6523687 hASPS_ASPSCR1-TFE3_DMSO, GSM6523688 hASPS_ASPSCR1-TFE3_JQ1, GSM6523689 hASPS_BRD4_DMSO, GSM6523690 hASPS_BRD4_JQ1
Genome browser session (e.g. UCSC)	UCSC

Methodology

Replicates	Two replicates
Sequencing depth	All samples were sequenced swingle-end 150 np with 10-30 million reads per sample on Illumina's MiSeq.
Antibodies	ASPSCR1 (Sigma-Aldrich, HPA026749), Histone H3K27ac (Active Motif, 39133), Histone H3K4me3 (Abcam, ab8580), Histone H3K27me3 (Millipore, 07-449),<br>FLAG (Sigma-Aldrich, F7425), and BRD4 (Bethyl Laboratories, A301-985A100)
Peak calling parameters	Peak calling was performed using MACS1.4 (http://liulab.dfci.harvard.edu/MACS) with a q-values (FDR) threshold of 0.01.
Data quality	Data quality was confirmed by the FastQC quality control tool. Duplicate ChIP-seq reads or reads with were removed with Samtools.
Software	Bowtie v2, Samtools v1.2, MACS v1.4, Cistrome (http://cistrome.org/ap/root), Nucleus (https://nas.rhelixa.com), NGSplot (https://anaconda.org/bioconda/r-ngsplot), IGV v2.3.80, HOMER v4.11.1, ROSE (http://younglab.wi.mit.edu/super_enhancer_code.html), GREAT v4.0.4.
